# Inhibition of incipient caries lesion progression by different fluoridated varnishes

**DOI:** 10.1590/0103-6440202405616

**Published:** 2024-05-10

**Authors:** Marcela Paris Mainente, Paula Andery Naves, Priscila Hernández de Campos, Marcela Charantola Rodrigues, Michele Baffi Diniz, Wanessa Christine de Souza Zaroni, Cristiane de Almeida Baldini Cardoso

**Affiliations:** 1 Graduate Program in Dentistry, Cruzeiro do Sul University. São Paulo-SP, Brazil.; 2 Department of Biomaterials and Oral Biology, School of Dentistry, University of São Paulo, São Paulo, Brazil.; 3 Department of Dentistry, University of Sorocaba, São Paulo, Brazil.

**Keywords:** Dental Caries, Tooth Demineralization, tooth remineralization

## Abstract

The aim of this in vitro study was to evaluate the potential of different fluoridated varnishes to inhibit the progression of incipient caries lesions after cariogenic challenge. Seventy-five enamel specimens of bovine teeth were prepared and selected based on the initial surface microhardness (SMH). The specimens were first subjected to artificial demineralization (in buffer solution) after which SMH was re-analyzed (SM1). They were then randomly assigned to five experimental groups: 1- CONTROL (pH cycling), 2 - MI VAR (MI Varnish with RECALDENT^TM^ - CPP-ACP), 3 - PROFL (Profluorid®), 4 - CLIN (ClinproTM White Varnish with TCP), and 5 - DUR (Duraphat®) (n=15). The varnishes were applied in a thin layer and the specimens were then subjected to pH cycling for eight days. The SMH and cross-sectional microhardness (CSMH) were then analyzed (SM2). The fluoride and calcium ion concentrations in the solution were analyzed by the indirect method and atomic absorption spectrophotometry, respectively. Data were statistically analyzed by Student’s t-test, ANOVA/Tukey-Kramer, or Kruskall-Wallis/Dunn tests for individual comparisons (p˂0.05). All varnishes led to significantly higher surface and subsurface remineralization compared with the control group but did not differ from each other. The varnishes with the highest fluoride release were: PROFL and CLIN, followed by MI VAR and DUR. The varnishes with significantly higher release of calcium were: DUR, CLIN, and PROFL. In conclusion, all commercial fluoridated varnishes tested have good potential to inhibit the progression of demineralization, regardless of the ion release mechanisms.

## Resumo

O objetivo deste estudo in vitro foi avaliar o potencial de diferentes vernizes fluoretados em inibir a progressão de lesões de cárie incipientes. Setenta e cinco espécimes de esmalte de dentes bovinos foram preparados e selecionados com base na microdureza superficial inicial (MDS/SM). Os espécimes foram submetidos à desmineralização artificial (em solução de Buskes), sendo então a MDS (SM1) reanalisada. Foram então distribuídos aleatoriamente em cinco grupos experimentais: 1- CONTROLE (pH cycling), 2 - MI VAR (MI Varnish with RECALDENT^TM^ - CPP-ACP), 3 - PROFL (Profluorid^®^), 4 - CLIN (Clinpro^TM^ White Varnish with TCP), and 5 - DUR (Duraphat^®^). Os vernizes foram aplicados em camada fina e os espécimes foram então submetidos à ciclagem de pH por oito dias. A MDS final (SM2) e a microdureza transversal (CSMH) foram então analisadas. As concentrações de flúor e íons cálcio em solução foram analisadas pelo método indireto e espectrofotometria de absorção atômica, respectivamente. Os dados foram analisados estatisticamente pelo teste T de Student, ANOVA/Tukey-Kramer ou testes de Kruskall-Wallis/Dunn para comparações individuais (p˂0,05). Todos os vernizes levaram a uma remineralização superficial e subsuperficial significativamente maior em comparação com o grupo controle, não diferindo significativamente entre si. Os vernizes com maior liberação de flúor foram: PROFL e CLIN, seguidos de MI VAR e DUR. Os vernizes com liberação significativamente maior de cálcio foram: DUR, CLIN e PROFL. Pôde-se concluir que todos os vernizes fluoretados comerciais testados apresentam bom potencial de inibição da progressão da desmineralização, independentemente dos mecanismos de liberação de íons.

## Introduction

The process of caries development is dynamic and multifactorial. It is considered to be a dysbiosis, as it results from the imbalance of interactions between the tooth surface, the microbial biofilm that forms on it (which is also present in normal situations), and sugars from food, in addition to other factors such as salivary flow and oral hygiene habits ^1^. The caries lesion is caused by the chemical dissolution of the tooth structure due to pH fluctuations and its first clinical sign is the appearance of white spots, which can be seen after tooth prophylaxis and air drying ^1^ ^,^ ^2^.

For adequate management, the activity of the caries lesion must be evaluated ^2^. The active white spot lesion is characterized by opaque enamel that feels rough to the touch of the probe and is often found in areas where biofilm is retained ^3^. If the lesion is detected at this stage, it is possible to perform a non-invasive treatment to inactivate the lesion, which is distinguished from the active lesion by the smooth and shiny appearance of the enamel ^1^.

Topical fluoride application is one of the most commonly used treatments for active incipient caries lesions, as it paralyzes and reverses mineral loss from the tooth structure. There are several ways to apply fluoride, such as toothpaste, gels, foams, mouthwashes, and varnishes ^1^. Fluoride varnish has been used in caries treatment and prevention for about 30 years. This product has a high fluoride concentration that promotes the formation of calcium fluoride on the tooth surface, which acts as a reservoir for the slow release of fluoride at sites at risk of caries or incipient caries ^4^.

Duraphat^®^ fluoride varnish (Colgate-Palmolive) has been considered the “gold standard” in dentistry and contains 5% sodium fluoride (22,600 ppm F) as the active ingredient. There are numerous studies in the literature showing its efficacy. A systematic review with meta-analysis including eight studies found a 38% reduction in caries lesions with the use of this product ^5^ ^,^ ^6^.

Several other varnishes have been launched with new technologies aimed at preventing the development of incipient lesions and inactivating existing caries lesions. Among them, varnishes containing fumaric acid-modified tricalcium phosphate (fTCP), amorphous calcium phosphate (ACP), and casein phosphopeptide-amorphous calcium phosphate (CPP-ACP) ^7^ are highlighted. The mechanism of action of these products is the release of calcium, phosphate, and fluoride ions.

Clinpro^TM^ White Varnish, launched by 3M ESPE ^8^, contains 2.23% fluoride and has TCP, the crystalline system of tricalcium phosphate, as a differential. This system has the potential to release calcium and phosphorus ions for the remineralization of incipient caries lesions, and the tricalcium phosphate particles are functionalized by being ground together with sodium lauryl sulfate ^9^. MI Varnish is a product of GC America, composed of 5% sodium fluoride and casein phosphopeptides-amorphous calcium phosphate (Recaldent: CPP-ACP) ^9^. Amorphous calcium phosphate (ACP) has the advantage of providing calcium and phosphate ions in amorphous form. However, the crystalline precipitation of these ions forms calculus and ACP does not adhere to the tooth surface. To solve these issues, casein phosphopeptide (CPP, a milk-derived nano complex) was added, ^10^ which binds to calcium and phosphate ions, preventing the formation of crystals. At a lower pH, chemical destabilization occurs, and CPP releases calcium and phosphate ions, allowing remineralization of the tooth surface to occur ^9^ ^,^ ^11^.

Finally, Profluorid^®^ varnish is a new product composed of 5% sodium fluoride and xylitol. Although it is indicated for the treatment of dentin hypersensitivity, there are still few studies in the literature evaluating its efficacy ^12^. The effect of xylitol against initial caries lesions has also been studied. Some authors have suggested that sorbitol and xylitol at very high concentrations in a saturated calcium sulfate solution form Ca_2_+-polyol complexes through the formation of cis-cis-triol coordination complexes. Based on these findings, the authors suggested that these polyols may influence calcium bioavailability in saliva and thereby directly promote the remineralization of deeper layers of demineralized enamel ^13^ ^,^ ^14^ ^,^ ^15^. However, the manufacturer does not report the concentration of xylitol added to the varnish. Since the best results of previous studies were obtained with 20% xylitol in varnishes ^16^ ^,^ ^17^ ^,^ ^18^ ^,^ ^19^ or 10% xylitol in toothpaste ^20^, it is not possible to say whether the effect of xylitol added to Profluorid™ is only favoring the taste of the varnish or if it could also be therapeutic.

Thus, this study aimed to evaluate the potential of fluoridated varnishes (Duraphat^®^, Clinpro^TM^ White Varnish with TCP, MI Varnish with RECALDENT^TM^ - CPP-ACP, and Profluorid^®^) in inhibiting the progression of demineralization in bovine teeth subjected to a new cariogenic challenge. The null hypothesis is that the different fluoride varnishes show no difference in inhibiting the progression of demineralization in bovine enamel.

## Materials and methods

### Specimen preparation

A total of 120 healthy bovine incisors without cracks or enamel defects were used. The dental crowns were separated from the roots using a precision metallographic cutter (Isomet 1000, Buehler Ltd., Lake Bluff, II, USA) with a diamond disk under refrigeration. A total of 183 enamel blocks measuring 4 x 4 mm were obtained from the flattest part of the buccal surface of each tooth.

Each enamel block was flattened with 600 and 1200-grit sandpaper and then polished with felt discs with diamond pastes (Teclago, Vargem Grande Paulista, São Paulo, Brazil) in grits of 6, 3, and 1 µm.

The surface microhardness (SMH) of each specimen was analyzed using a digital microhardness tester with a Knoop indenter (HMV-2T, Shimadzu, Kyoto, Japan). Five indentations were performed in the center of each specimen at a spacing of 100 µm with a static load of 25 g/f for 10 seconds ^17^. Seventy-five specimens were selected by calculating the mean and standard deviation of microhardness.

### Initial demineralization

A layer of acid-resistant nail polish was applied to half of the surface of each enamel block (8 mm^2^), which was used as a control area for each specimen. For the formation of the artificial caries lesion, each specimen was maintained in 40 mL (5 mL solution/mm^2^ of exposed enamel) of 0.05 M acetate buffer solution, 1.28 mM Ca (Ca (NO_3_)_2_.4H_2_O), 0.74 mM P (KH_2_PO_4_) and 0.03 micrograms F/mL (NaF), pH 5.0, for 48 hours at 37°C ^21^.

After that, a new SMH analysis was performed (SM1), using the same steps described above.

### Experimental groups

The demineralized enamel blocks were randomly distributed into five groups of 15 specimens each, as shown in Box 1. Groups 2 - MI VAR (MI Varnish with RECALDENT^TM^ - CPP-ACP), 3 - PROFL (Profluorid^®^), 4 - CLIN (Clinpro^TM^ White Varnish with TCP), and 5 - DUR (Duraphat^®^) were submitted to treatment with fluoride varnish according to the recommendations of each manufacturer.

The different varnishes were applied to the enamel blocks with a microbrush, and the blocks were then individually stored in 30 mL of artificial saliva for 6 h ^18^. At the end of this period, the varnish was carefully removed using a scalpel blade, without touching the enamel surface ^17^ ^,^ ^18^.

**Box 1:**
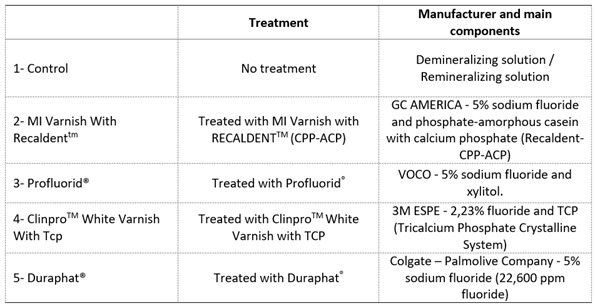
Experimental groups

### pH cycling

An eight-day pH cycling regime was used, in which the blocks remained in demineralizing solution (1.28 mM Ca, 0.74 mM P, 0.03µg F/mL, pH 5.0) for 2 h and in remineralizing solution (1.5 mM Ca, 0.13 mM P, 150 mM KCl, 0.05 µg F/mL, 0.1 M Tris solution, pH 7.0) for 22 h, at 37˚C ^21^. Before and after immersion in the demineralizing solution, the blocks were washed with deionized and distilled water.

The solution volume per block area was kept constant at 6.25 mL/mm^2^ for the demineralizing and 3.12 mL/mm^2^ for the remineralizing solution. In the fourth cycle, the two solutions were exchanged and after the eighth cycle, the specimens were evaluated ^21^.

### Final surface microhardness analysis

After pH cycling, the SMH (SM1) was reassessed, following the same steps described previously.

### Final subsurface (cross-sectional) microhardness analysis

For cross-sectional hardness (CSH) testing, the blocks were cut longitudinally down the center, embedded, and polished. Two rows of eight indentations - one in the central region of exposed enamel and the other in the control area (protected with nail polish) - were made with a 25-g load for 10 s. The indentations were made at 10, 30, 50, 70, 90, 110, and 220 μm from the outer enamel surface in two sequences. The mean values of the two measurement points, each at 100 μm from the surface, were then averaged. The integrated area under the curve (cross-sectional hardness profiles into the enamel) using hardness values (KHN) was calculated by the trapezoidal rule for each depth (μm) from the lesion to the sound enamel. This value was subtracted from the integrated area of sound enamel to obtain the integrated area of subsurface enamel; this was named the integrated loss of subsurface hardness (ΔKHN) ^22^.

### Fluoride and calcium release analysis

Fluoride and calcium release were done on the cycling solutions, with a sum of solutions at the end of the fourth and eighth days of pH cycling. Fluoride ion concentration in solution was analyzed by the indirect method, using an ion-specific electrode (Thermo ORION 9609, Beverly, MA, USA), with a detection limit of 0.02 mg/L, linked to a potentiometer (Thermo ORION 720A+, Beverly, MA, USA). Standard solutions with fluoride concentrations ranging from 0.005 to 0.19 µg were used, and only calibration curves with a percentage variation of up to 5% for all standards and r ≥ 0.99 (linearity) were accepted. The calibration curve and experimental samples were buffered with the same volume of standard total ionic strength adjustment buffer solution (TISAB II) according to the method of Taves (1968) ^23^ modified by Whitford (1996) ^24^. Readings were performed in duplicate on 1 mL of each sample, obtaining values in mV, which were later converted into µg/mL. The average of the two readings was used as the result for each sample.

The release of calcium ions was measured in an atomic absorption spectrophotometer with a specific calcium cathode lamp, at a wavelength of 422.70 nm. Standard solutions ranging from 5 to 80 mg/L were used. Lanthanum chloride solutions were used to minimize distortions in the readings. The calcium released from the samples was analyzed by the colorimetric method. To do this, 50 µL of d-H_2_O was added to the wells of a microplate (96 wells), then 1 µL of the sample followed by 50 µL of Arsenazo III. The reference value used was from the reagent kit used for the analysis, ranging from 8.5 to 10.5 mg/dL ^25^.

### Statistical analysis

Data analysis was performed using BioEstat (5.3). The significance level was set at 5% for all analyses. The data of surface microhardness at baseline (SM0), after induction of artificial caries lesion (SM1), and after pH cycling (SM2) for the experimental groups were submitted to the Kolmogorov-Smirnov test to verify normality and then submitted to one-way analysis of variance (ANOVA) followed by Tukey's test. Paired Student t-test was performed to compare SM1 versus SM2 (Table 1).

**Table 1:** Mean and standard deviation of surface microhardness at baseline (SM0), after induction of artificial caries lesion (SM1), and after pH cycling (SM2) for the experimental groups.

Group	SM0	SM1	SM2	p-value*
1- Control	337.8 ± 59.6 ^A^	187.6 ± 35.2 ^A.a^	140.2 ± 54.0 ^A,b^	0.0007
2 - Mi Varnish	328.9 ± 54.9 ^A^	203.7 ± 35.7 ^A.a^	220.9 ± 38.2 ^B.a^	0.1344
3 - Profluorid	322.9 ± 53.6 ^A^	180.3 ± 46.0 ^A.a^	201.3 ± 46.7 ^B.a^	0.2602
4 - Clinpro	338.0 ± 43.0 ^A^	179.4 ± 35.2 ^A.a^	203.6 ± 56.9 ^B.a^	0.1800
5 - Duraphat	327.6 ± 61.2 ^A^	210.5 ± 42.8 ^A.a^	204.4 ± 64.0 ^B.a^	0.7158
p-value**	0.942	0.168	0.001	

*Paired Student t-test (SM1 versus SM2)

**One-way ANOVA

Different uppercase letters indicate a statistically significant difference within the same column (p<0.05)

Different lowercase letters indicate a statistically significant difference within the same row (p<0.05)

Fluoride and calcium ion concentrations in the demineralization (DE) and remineralization (RE) solutions for the experimental groups were submitted to the Kolmogorov-Smirnov test to verify normality and then submitted to one-way analysis of variance (ANOVA) followed by Tukey-Kramer test. Fluoride ion concentration in the remineralization solution was submitted to Kruskal-Wallis, followed by Dunn’s test for individual comparisons (Table 2).

**Table 2:** Mean and standard deviation of fluoride and calcium ion concentrations in the demineralization (DE) and remineralization (RE) solutions for the experimental groups.

Group	Fluoride (µg/g)		Calcium (mg/L)	
	DE	RE	DE	RE
1- Control	0.11 ± 0.03 ^A^	0.11 ± 0.01 ^A^	14.9 ± 6.7 ^A^	20.8 ± 2.1 ^A^
2 - MI Varnish	0.19 ± 0.05 ^B^	0.10 ± 0.02 ^A^	19.9 ± 2.5 ^B^	22.9 ± 1.8 ^A^
3- Profluorid	0.25 ± 0.04 ^C^	0.09 ± 0.04 ^B^	21.4 ± 4.1 ^B, C^	20.7 ± 2.9 ^A^
4 - Clinpro	0.26 ± 0.08 ^C^	0.15 ± 0.07 ^C^	21.9 ± 6.0 ^c^	20.7 ± 2.2 ^A^
5 - Duraphat	0.19 ± 0.07 ^B^	0.11 ± 0.08 ^A^	25.1 ± 4.7 ^D^	22.6 ± 3.3 ^A^
p-value**	<0.001*	0.000830**	0.006*	0.180*

*One-way ANOVA, Tukey-Kramer post test

**Kruskal-Wallis, Dunn post-test

Significant differences are indicated by different uppercase letters within the same column (p<0.05)

Mean and standard deviation of integrated mineral loss values, calculated from cross-sectional microhardness values for the experimental groups were submitted to the Kolmogorov-Smirnov test to verify normality and then submitted to Kruskal-Wallis, followed by Dunn’s test for individual comparisons (Table 3).

**Table 3:** Mean and standard deviation of integrated mineral loss values, calculated from cross-sectional microhardness values for the experimental groups.

Group	ΔKHN (95% CI)
1- Control	4008±5520^b^
2 - MI Varnish	1634±903^a^
3 - Profluorid	1617±1512^a^
4 - Clinpro	1188±707^a^
5 - Duraphat	1001±821^a^
p-value*	0.016

*Kruskal-Wallis followed by Dunn's test (p <0,05). Different lowercase letters indicate a statistically significant difference within the same row (p<0.05).

## Results

All tested fluoridated varnishes had a significantly higher surface and subsurface remineralization capacity compared to the control group and did not differ from each other (Tables 1 and 3).

The varnishes with the highest release of fluoride ions in the demineralizing solution were PROFL and CLIN, followed by MI VAR and DUR. In the remineralizing solution, CLIN released the highest amount of fluoride ions, and PROFL, the lowest amount. The varnish with the highest release of calcium ions in the demineralizing solution was DUR, followed by CLIN, and PROFL. In the remineralizing solution, there was no statistically significant difference between the studied varnishes (Table 2).

## Discussion

Considering the wide applicability and advantages of fluoridated varnish, its clinical application is extremely important. With technological advances and the introduction of new fluoride varnishes with innovations, there is an increasing need for studies that evaluate the effectiveness of these products and indicate which products should be the first choice.

Duraphat^®^ fluoride varnish has always been considered the “gold standard” varnish ^6^ in dentistry, but some studies found no difference between the remineralization potential of Duraphat^®^, Clinpro^TM^ White (TCP), and Enamel Pro^TM^ (ACP) Duraphat^® (^ ^26^. The results of the present study show no significant difference between other commercial fluoridated varnishes and Duraphat^®^ in inhibiting the progression of incipient caries lesions. In addition, Duraphat^®^ has a yellowish color, which is a limitation in its use, especially for adult patients. The other varnishes are colorless and are preferred from an aesthetic point of view.

A previous study that used cumulative chromatography methods (µmol per gram) found that MI Varnish containing CPP-ACP had higher calcium and phosphate ion release than Clinpro^TM^ White containing TCP and Duraphat^®^, which did not differ from each other ^7^. These results are not consistent with our findings on calcium ion release analyzed by spectrophotometry, in which Duraphat^®^, followed by Clinpro^TM^ White and Profluorid^®^ varnishes, had the highest calcium release in the demineralization solution.

Therefore, it seems that there are different ionic interactions among varnish, substrate, and solution and different concentrations of ions in the solution, depending on the analyzed ion and analyzed solution (demineralization or remineralization), which in the end may not directly influence the effectiveness of the varnish. Based on the results of the present study, different compositions and in-solution interactions of varnishes may modulate demineralization and remineralization in different ways but with similar effectiveness. This was confirmed by the inhibition of caries progression evaluated by surface and longitudinal microhardness.

Studies show that the difference in ion release kinetics is due to variations in the chemical composition of the analyzed materials (additional compounds). A study comparing 3 varnishes with different compounds (CPP-ACP, BAG (bioactive glass), and fluoride) found that the release rate was different. The MI varnish (CPP-ACP) had a higher F release in the first 6 hours and the highest release after 48 hours. The other 2 varnishes had a slower ion release, due to the characteristics of the bioactive glass and the F varnish itself ^27^. In this study, the MI Varnish also had a higher initial release of Ca and phosphorus ions compared to the other varnishes.

Fluoride associated with calcium and phosphate ions can inhibit the demineralization process of teeth by replacing the hydroxyl ion of hydroxyapatite with the fluoride ion and forming fluorapatite. In this way, the newly formed crystals make the tooth structure more resistant to demineralization. Even with new technologies such as bioactive glass and other materials considered bioactive, the results of recent studies show that the main mechanism of action of varnishes is to make fluoride bioavailable in addition to calcium and phosphate. The varnish containing CPP-ACP released more ions (Ca, F, and P) than the bioactive glass varnish, while the fluoride varnish seemed to retain fluoride longer during the study period ^27^.

The chemical composition of the inactive ingredients and/or the calcium phosphate system influences ion release dynamics, independent of the fluoride content. Regardless of whether the release time is short or long, this release is of clinical importance, and these materials can potentially improve the remineralization process in both enamel and dentin, which was observed in the present *in vitro* study.

Another study by Godoi *et al* (2019) ^28^ evaluated the concentrations of soluble and insoluble fluoride in commercial varnishes and their effect on the remineralization of artificial caries lesions in enamel by superficial and cross-sectional microhardness evaluations. Four varnishes were tested: control (no treatment), Enamelast (Ultradent Products), Duraphat^®^ (Colgate-Palmolive), and Clinpro White^TM^ Varnish (3M ESPE). The authors also performed polarized light microscopy to analyze subsurface caries lesions. The results indicated that the Enamelast and Duraphat^®^ varnishes presented significantly higher enamel microhardness than the control and Clinpro White^TM^ groups. This differs from the results of the present study, in which all-commercial varnishes, including Clinpro White^TM^, were able to inhibit the progression of demineralization after a second cariogenic challenge (pH cycling).

Polarized light microscopy images in the study by Godoi *et al.* (2019) ^28^ ^)^ showed that the subsurface carious lesions were not remineralized by the varnish treatments and no significant differences in depth microhardness were observed between treatments. These results are partially consistent with the longitudinal hardness results of the present study, as there were no significant differences among the commercial varnishes, but all had significantly higher remineralization capacity of the subsurface lesion compared with the control varnish. However, remineralization was observed up to 100 micrometers in longitudinal hardness analysis, which may not correspond to the complete remineralization of the white spot in a clinical situation, which should be confirmed by properly designed randomized controlled clinical trials with commercial varnishes.

These findings must be confirmed in models that had better resemble reality, such as *in situ* studies or microcosm biofilm models, since *in vitro* models, such as pH cycling, occur in a “closed” system where mineral exchange between enamel and the demineralizing and remineralizing solutions is limited by the ionic balance between them. However, clinical trials are expensive and time-consuming, and some varnishes may lead to dental pigmentation, compromising acceptability and compliance to the study, which justifies testing these products previously *in vitro* models.

## Conclusion

All commercially available fluoridated varnishes tested inhibited the progression of demineralization, regardless of the mechanism of action or concentration of ions released.
